# Longitudinal data for magnetic susceptibility of normative human brain development and aging over the lifespan

**DOI:** 10.1016/j.dib.2018.06.005

**Published:** 2018-06-15

**Authors:** Yuyao Zhang, Hongjiang Wei, Matthew J. Cronin, Naying He, Fuhua Yan, Chunlei Liu

**Affiliations:** a Electrical Engineering and Computer Science, University of California at Berkeley, CA, USA; b Department of Radiology, Ruijin Hospital, Shanghai Jiaotong University School of Medicine, Shanghai, China; c Helen Wills Neuroscience Institute, University of California at Berkeley, CA, USA

## Abstract

The data presented in this article accompany the research article entitled “Longitudinal Atlas for Normative Human Brain Development and Aging over the Lifespan using Quantitative Susceptibility Mapping” (Zhang et al., 2018) [1]. The longitudinal evolution of magnetic susceptibility in human brain indicates critical characteristics of normal brain development and aging. In the corresponding research article, we build longitudinal QSM atlases over various age intervals using 166 healthy subjects (83F/69M) with an age range of 1–83 years old. Based on the newly built atlases, we investigate the regional evolutions of magnetic susceptibility in the brain. In this article, we report anatomical evolutions of the age-specific QSM atlases in deep gray matter nuclei and in two selected white matter fiber bundles. In addition to iron-rich brain nuclei, the evolution patterns of the magnetic susceptibility in the amygdala and hippocampus are also presented.

**Specifications Table**

**Table:** 

Subject area	*NeuroImaging*
More specific subject area	*MRI, Quantitative Susceptibility Mapping, Image analysis*
Type of data	*Figures*
How data was acquired	Three-dimensional multi-echo gradient echo (GRE) MRI sequence was utilized to obtain T2*-weighted images
Data format	*Analyzed*
Experimental factors	*Data from 8 infant subjects (age 1–2 years, 4M/4F), 22 children (age 3–10 years, 8M/14F) subjects, 19 teenage (age 11–20 years, 10M/9F) subjects, 45 younger adult (age 22–53 years, 22M/23F) subjects and the 72 older adult (age 46–83 years, 30M/42F) subjects were used in the analysis.*
Experimental features	*The QSM images were first segmented to 204 brain regions with automated atlas-based image segmentation, and then the susceptibility from each region were used for susceptibility evolution model study.*
Data source location	*Brain Imaging and Analysis Center (BIAC) at Duke University, Durham, NC, USA*
	*Rui Jin Hospital, Shanghai, China*
Data accessibility	*Data are within this article.*

**Value of the data**

• Anatomical evolutions of the age-specific QSM atlases in deep gray matter nuclei and in two selected white matter fiber bundles show delicate view of brain tissue development.
• Our automated atlas-based susceptibility evolution analysis provided a systematic and comprehensive confirmation of the previous findings on age-related iron accumulation resulting from manual ROI drawings.
• The susceptibility evolution in amygdala and hippocampus indicates continuous iron deposition with age.

## 1 Data

### 1.1 QSM(T1w) hybrid image generation

**Fig. 1:**
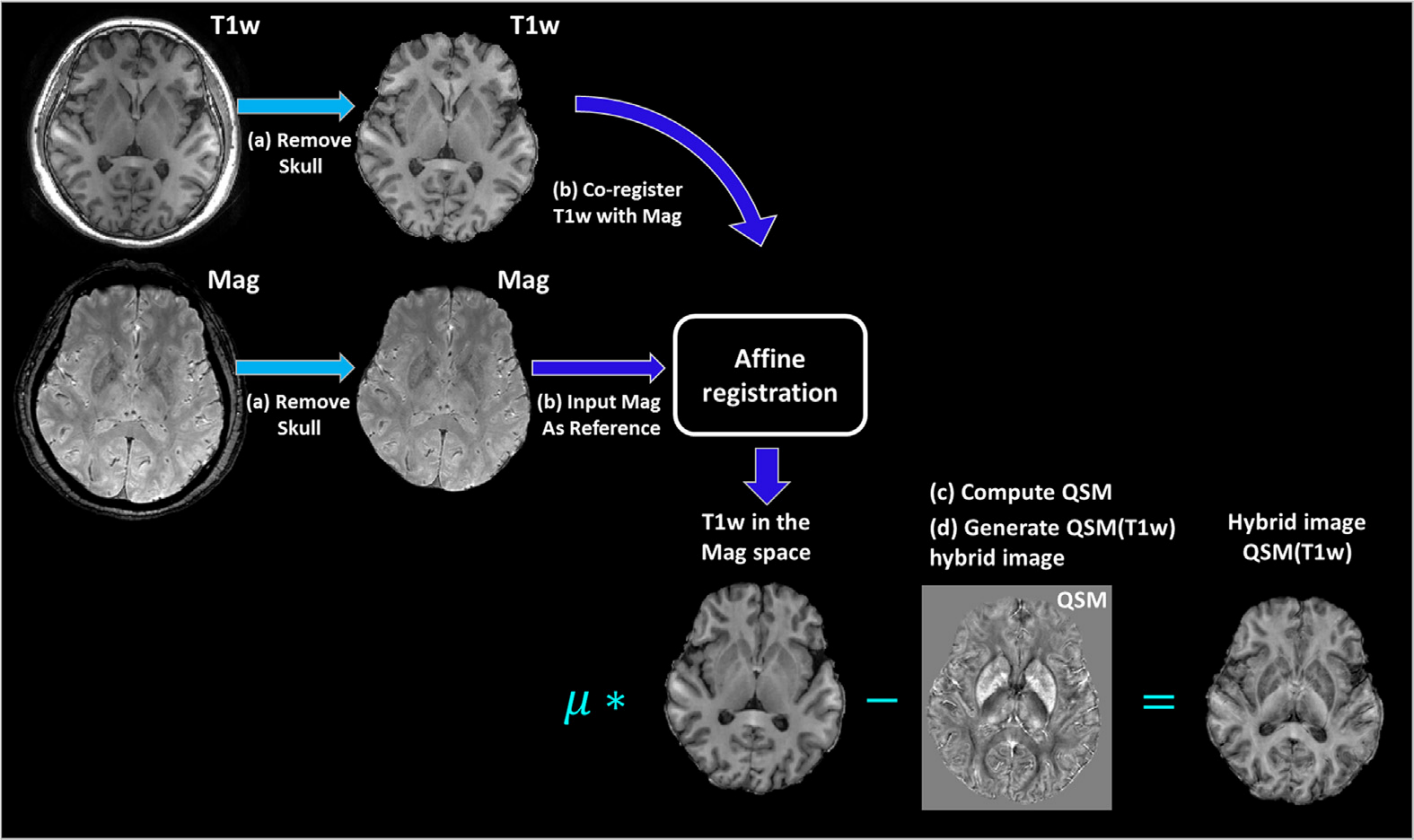
Flowchart for creating QSM(T1w) hybrid image.

Conventional T1-weighted (T1w) MRI presents high contrast between cortical gray and white matter, while quantitative susceptibility mapping provides high contrast among iron-rich deep brain nuclei and between deep gray and white matter (Hanspach al.,[8]. Therefore, in the related research paper [1], the two complementary contrasts are incorporated to generate a hybrid contrast to guide the registration for atlas construction. In Fig. 1, we demonstrate the process for creating the QSM(T1w) hybrid contrast. Firstly, the skull was removed from both the GRE magnitude image and the T1-weighted image using FSL BET (Smith et al. [4]) (Fig. 1(a)). Secondly, the T1-weighted images were co-registered to the corresponding magnitude images using FSL FLIRT (Smith et al. [4]) (Fig. 1(b)). Thus, the QSM images and T1-weighted image were in the same anatomical space. The intensity of T1-weighted image was normalized to be in the range of [0.255]. Lastly, the QSM(T1w) hybrid images were generated according to Eq. (1) in the research paper [1], here the scalar weighting variable μ is empirically set as 0.0025. As illustrated in Fig. 1(d), the hybrid image preserves both enhanced anatomical contrast of deep brain nuclei in the susceptibility map and clear cortical structures defined in the T1-weighted image.

### 1.2 Anatomical evolution of age-specific QSM atlases in deep gray matter nuclei

**Fig. 2:**
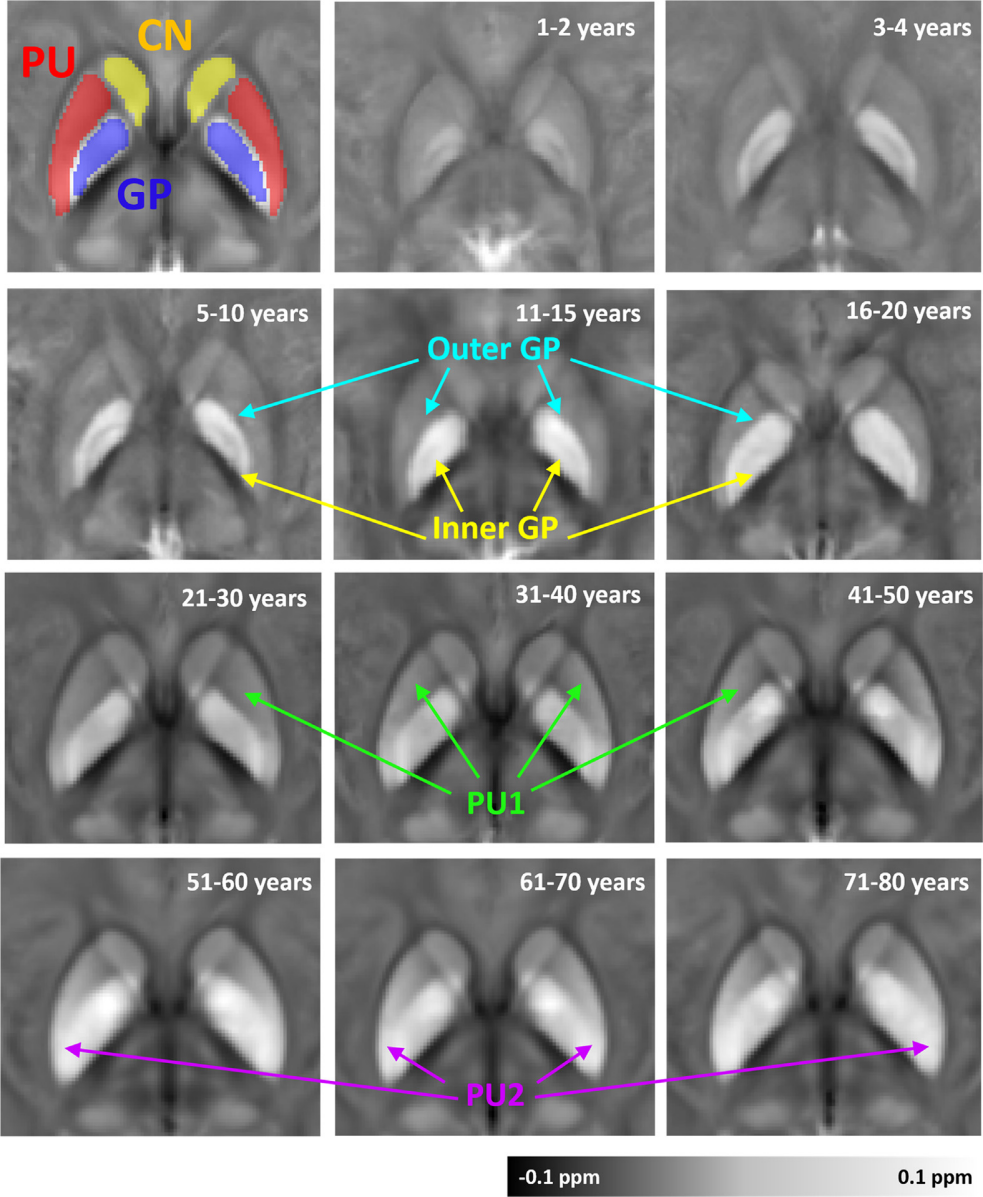
Anatomical evolution of age-specific susceptibility atlas with aging for the putamen (PU), globus pallidus (GP) and caudate nucleus (CN). The magnetic susceptibility of these deep gray matter regions increases significantly with age. Moreover, the changes in physical shape, volume and intra-regional heterogeneity with aging are revealed by magnetic susceptibility.

**Fig. 3:**
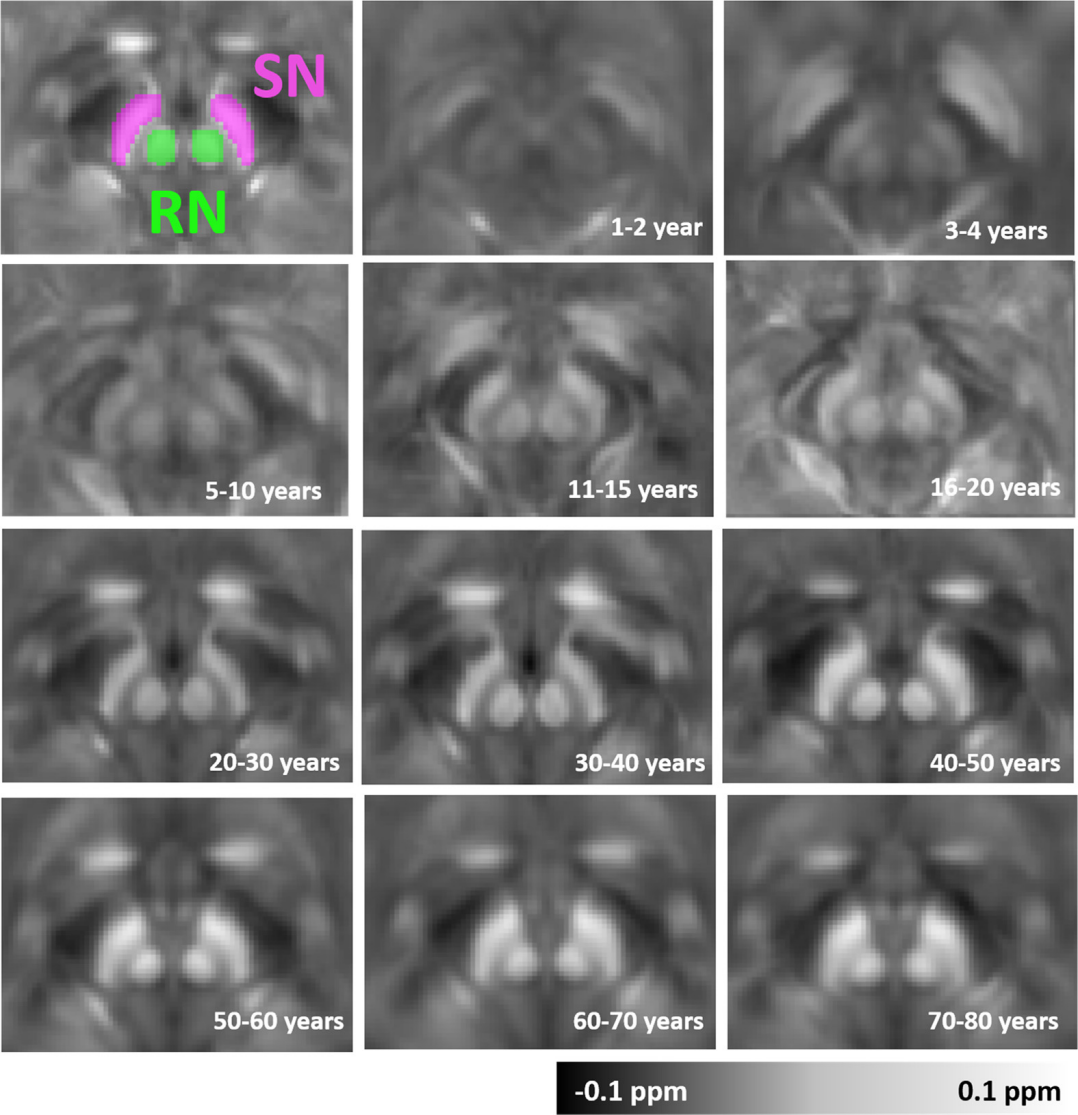
Anatomical evolution of age-specific susceptibility atlas with aging for the red nucleus (RN) and substantia nigra (SN). Magnetic susceptibility of these deep gray matter regions significantly increases with age. Moreover, changes in the distribution of magnetic susceptibility within the ROIs are shown by the magnetic susceptibility atlases.

**Fig. 4:**
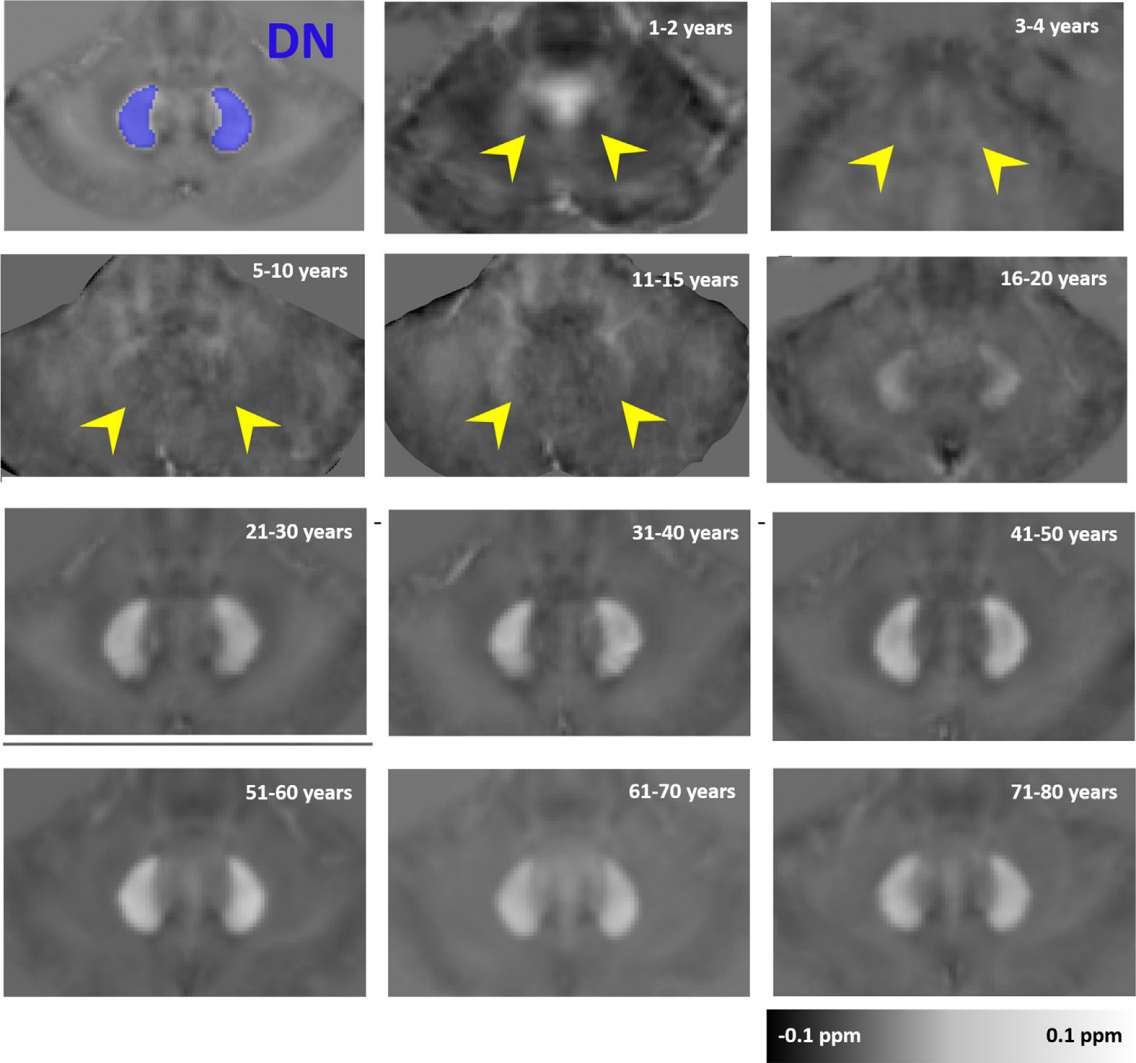
Anatomical evolution of age-specific susceptibility atlas with aging for the dentate nucleus (DN). Magnetic susceptibility of the DN increases significantly with age. Variation of the morphology and contrast with age are demonstrated here by susceptibility images.

Progressive accumulation of iron with aging has been well reported in brain tissues (Hallgren and Sourander [6]). As iron content is the main contributor to the bulk magnetic susceptibility in deep gray nuclei (Schweseg al.,[9], susceptibility in deep gray matter nuclei show rising contrast with aging. In the related research paper [1], authors built age-specific atlases for various age intervals over the lifespan. As shown in Fig. 2, close-up views of the putamen (PU), globus pallidus (GP) and caudate nucleus (CN) in each age-specific atlas are presented. Fig. 3 shows the enlarged RN and SN regions in the age-specific QSM atlases. Fig. 4 shows the enlarged DN regions in the age-specific QSM atlases.

**Fig. 5:**
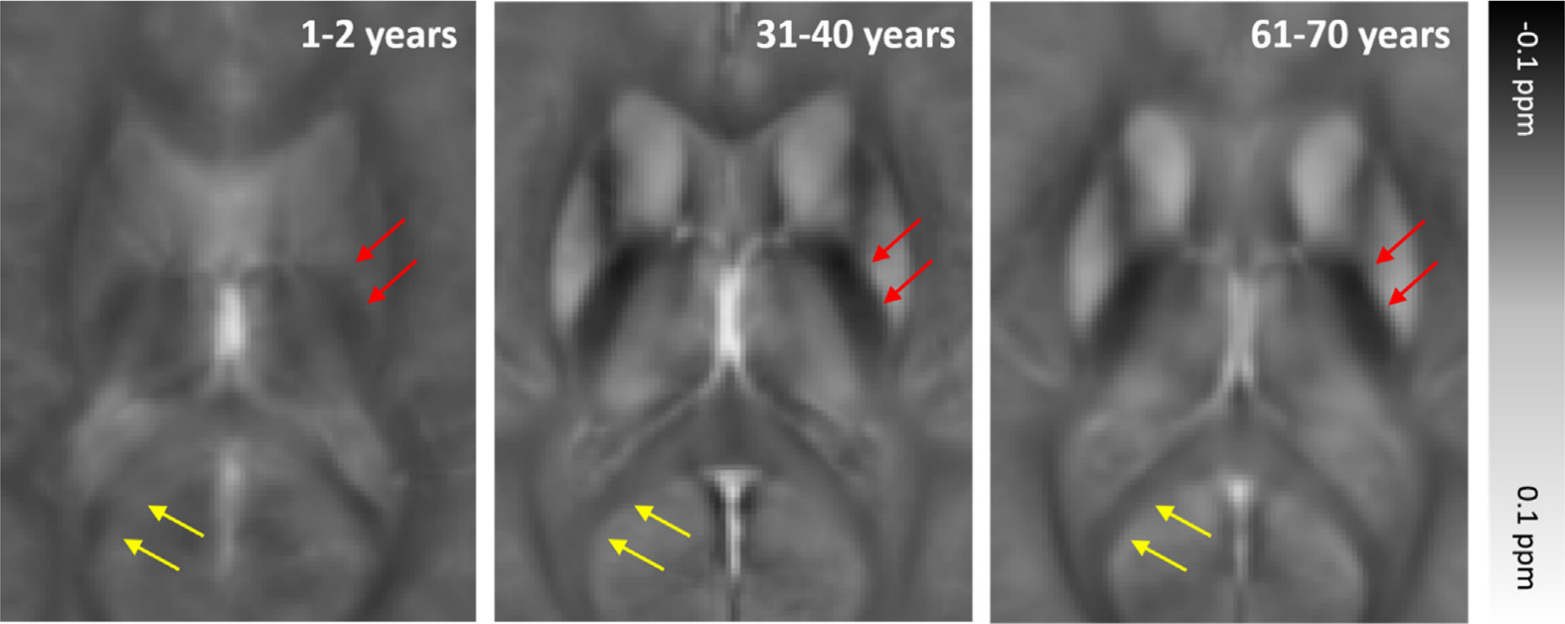
Anatomical evolution of age-specific susceptibility atlas with aging within selected white matter bundles: the posterior limb internal capsule (PLIC) indicated by red arrows; and splenium of corpus callosum (SCC) indicated by yellow arrows.

Fig. 5 in Ref. [1] shows the susceptibility evolution in two white matter bundles, the posterior limb of the internal capsule (PLIC) and the splenium of the corpus callosum (SCC). In the youngest brain atlas (1–2 years), the susceptibility contrast between white matter and gray matter is apparently lower than that of the middle-age brain template (31–40 years). The white matter bundles of the old-age template (61–70 years) become relatively more paramagnetic comparing to those of the 31–40 years-old template. This result indicates that the brain white matter myelinates during brain maturation and then demyelinates with aging which is consistent with previous DTI studies on normal aging [2] (Fig. 5).

### 1.3 Susceptibility evolution with age in deep gray matter nuclei, amygdala and hippocampus

Putamen (PU), globus pallidus (GP), substantia nigra (SN), red nucleus (RN), caudate nucleus (CN), dentate nucleus (DN), amygdala (AL) and hippocampus (HiP). Susceptibility values of these nuclei are fitted with the exponential growth pattern in Eq. (1) (in Section 2.3). The fitted equations are listed in Table 2 in Ref. [1].

**Fig. 6:**
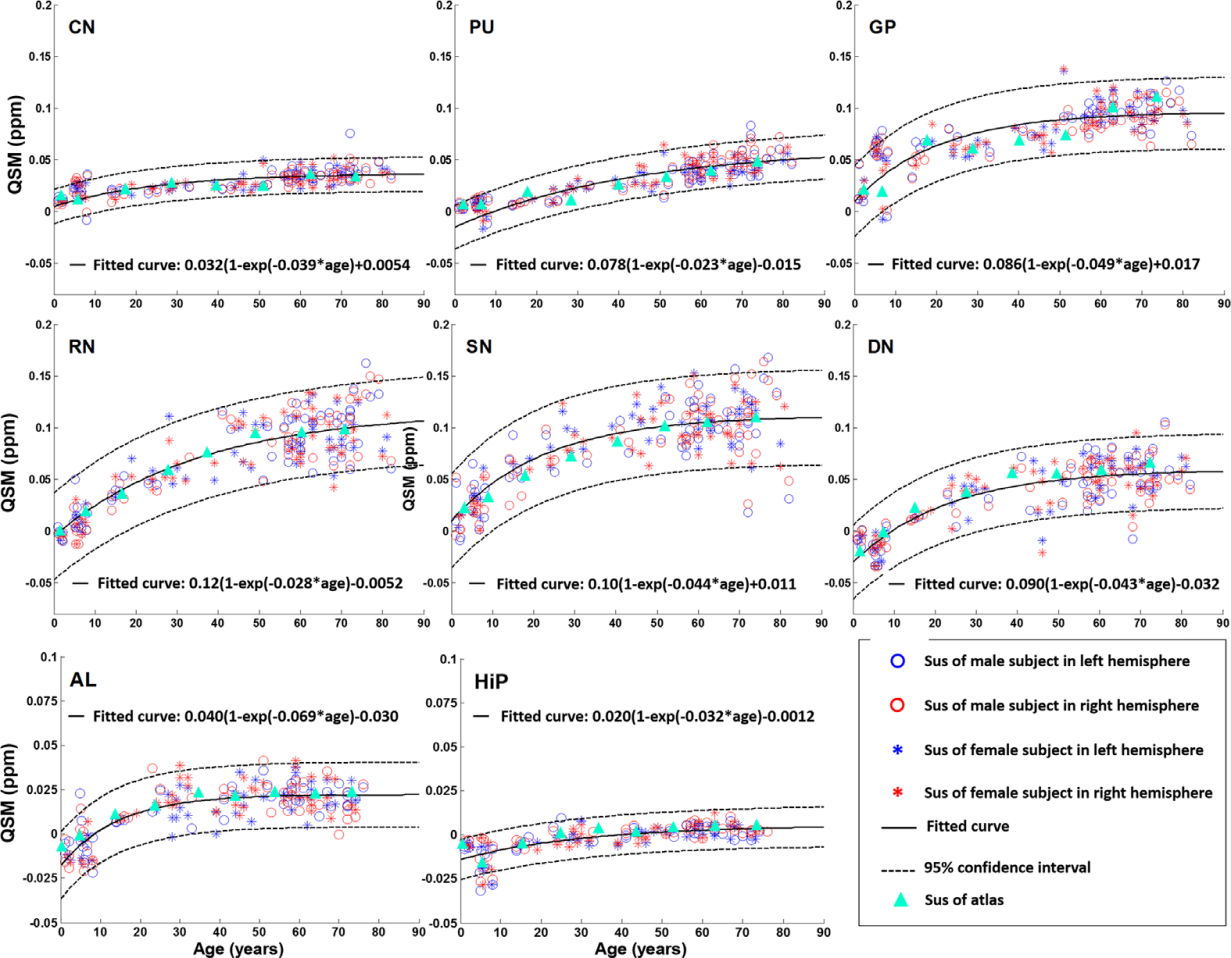
Susceptibility evolution with age in deep gray matter nuclei, amygdala and hippocampus.

We collected the average susceptibility value in eight ROIs (PU, CN, GP, SN, RN, DN, AL and HiP) from each individual subject (age 1–83 years), then fitted the collected aging data with the exponential model in Eq. (1) (in Section 2.3). The regression lines are shown in Fig. 6. The fitted model of mean susceptibility values for each ROIs are listed in Table 2 in Ref. [1].

### 1.4 Creation of infant brain parcellation map

**Fig. 7:**
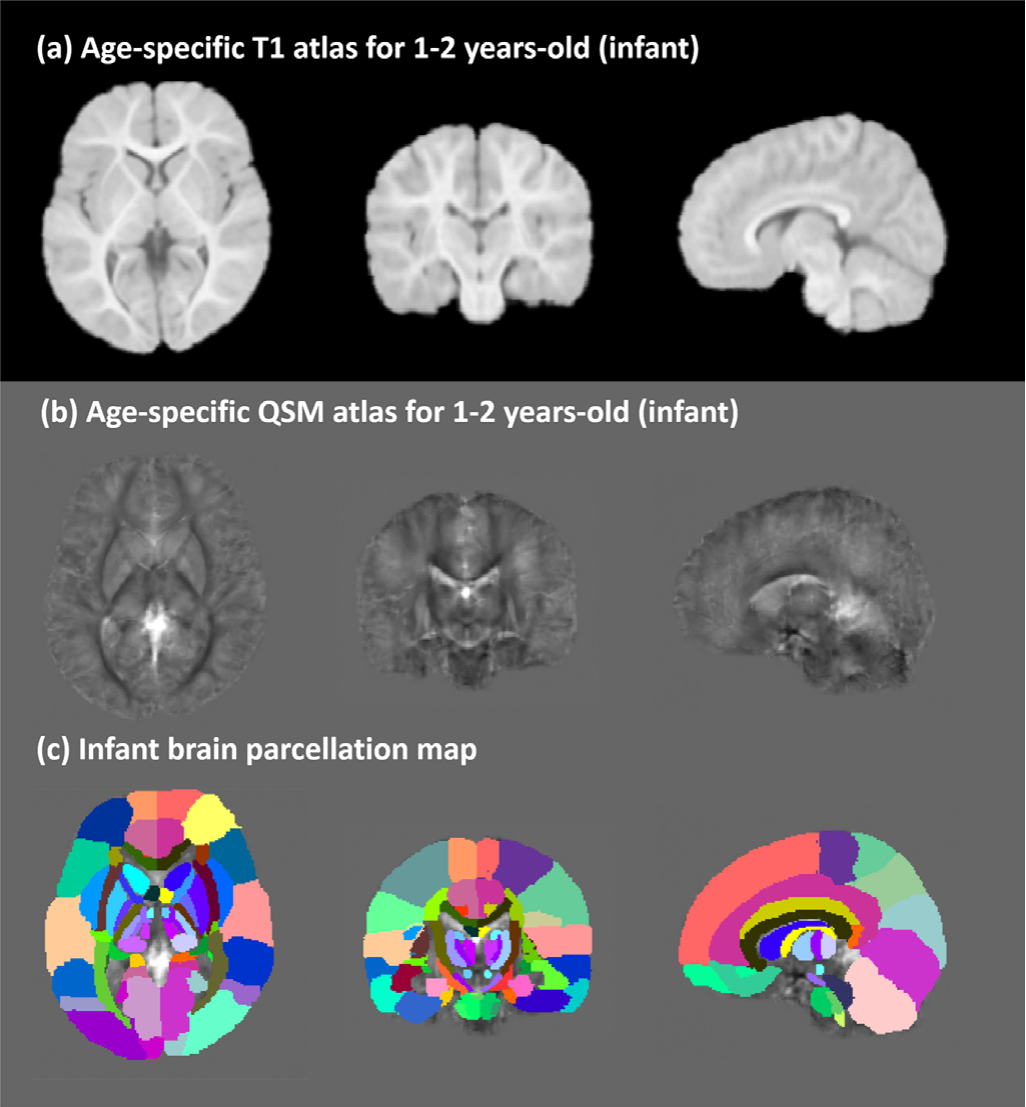
Performance of infant brain age-specific QSM atlas. (a) The T1-weighted infant atlas. (b) The QSM infant atlas. (c) The “infant whole brain QSM parcellation map”.

Infant brains are substantially different from adult brain in anatomically structure and imaging contrast. Therefore, the generation of infant “whole brain QSM parcellation map” is specialized. Firstly, the JHU T1-weighted infant atlas [3] was registered towards the proposed T1-weighted infant atlas (as shown in Fig. 7(a), which is consistent with our proposed infant QSM atlas) via FSL affine registration (FLIRT) (Smith et al. [4]) followed by diffeomorphic registration (DEMONS) (Vercauteren et al. [5]). The deformation fields were then applied to the 122 ROIs infant brain parcellation [3] to warp the cortical and white matter fiber bundle ROIs into the infant QSM atlas space. Furthermore, we removed 12 ROIs, including the caudate nucleus (CN), putamen (PU), globus pallidus (GP), hippocampus (HP), amygdala (AL) and thalamus (TL) from the warped infant parcellation map, and manually created these ROIs and the bilateral red nuclei (RN), substantia nigra (SN), dentate nucleus (DN) and sub-region nuclei of thalamus based on the proposed infant QSM atlas.

The proposed infant QSM atlas was shown in Fig. 7(b), and the consistent T1-weighted structure is illustrated in Fig. 7(a). The “infant whole brain parcellation map” is performed in Fig. 7(c).

## 2 Experimental design, materials and methods

### 2.1 Data acquisition and reconstruction

The infant, children and teenager subjects were scanned at the Brain Imaging and Analysis Center (BIAC) at Duke University, using a 3T scanner (MR 750, GE Healthcare, Milwaukee, WI). While the adult subjects were scanned at Rui Jin Hospital (Shanghai, China), using a 3T scanner (Signa HDxt, GE Healthcare, Milwaukee, WI). Conventional T1-weighted images with 1 mm isotropic resolution were acquired to display brain structure. Thereafter, a three-dimensional multi-echo gradient echo (GRE) sequence was utilized to obtain T2*-weighted images with the following scan parameters:

The 8 infant subjects (age 1–2 years, 4M/4F) were scanned using a GE MR750 3T with echo time (TE)=40 ms, repetition time (TR)=50 ms, and an original spatial resolution of 1×1×1 mm^3^. Infants were scanned without being sedated and were fed before scanning. Neonatal earmuffs were used for hearing protection, and possible motion artifacts were mitigated by immobilization with a cotton pillow. An experienced neonatologist and a neuroradiologist were in attendance throughout the imaging process. A pulse oximeter was used to monitor heart rate and oxygen saturation. The 22 children (age 2–10 years, 8M/14F) subjects were scanned using a GE MR750 3T TE1/spacing/TE8=5/2.94/25.6 ms, TR=55 ms, and an original spatial resolution of 0.6×0.6×1.5 mm^3^. The 19 teenage (age 11–20 years, 10M/9F) subjects were scanned using a GE MR750 3T scanner with TE1/spacing/TE8=4/2.82/29.4 ms, TR=41 ms, and an original spatial resolution of 0.86×0.86×2 mm^3^. The 45 younger (age 22–53 years, 22M/23F) subjects and the 72 older (age 46–83 years, 30M/42F) subjects were scanned on a GE Signa HDxt 3T scanner with TE1/spacing/TE8=5.468/3/26.5 ms, TR=54.6 ms, and an original spatial resolution of 0.86×0.86×2.0 mm^3^.

### 2.2 Atlas-based image segmentation

Using a linear transformation [4] followed by Demons registration [5],individual subjects are aligned to the longitudinal atlases proposed in paper [1]. Through this procedure, the brains were segmented to regions of interests (ROIs) automatically.

### 2.3 Fitting susceptibility evolution in the deep gray matter nuclei

Gradual deposition of iron with aging has been well reported in brain tissues [6]. The susceptibility evolution can be modeled using the exponential growth model [7], which was originally proposed by Hallgren et al. to model the iron concentration evolution:

(1) Sus[ppm]=α(1−exp(−β*age[y/o]))+γ

where scalar variables α, β and γ are tissue specific parameters, with β defining the rate of the exponential growth.
